# Gastrodin Protects Apoptotic Dopaminergic Neurons in a Toxin-Induced Parkinson's Disease Model

**DOI:** 10.1155/2013/514095

**Published:** 2013-03-03

**Authors:** Hemant Kumar, In-Su Kim, Sandeep Vasant More, Byung-Wook Kim, Young-Yil Bahk, Dong-Kug Choi

**Affiliations:** Department of Biotechnology, Konkuk University, Chungju 380-701, Republic of Korea

## Abstract

*Gastrodia elata* (GE) Blume is one of the most important traditional plants in Oriental countries and has been used for centuries to improve various conditions. The phenolic glucoside gastrodin is an active constituent of GE. The aim of this study was to investigate the neuroprotective role of gastrodin in 1-methyl-4-phenylpyridinium (MPP^+^)/1-methyl-4-phenyl-1,2,3,6-tetrahydropyridine- (MPTP) induced human dopaminergic SH-SY5Y cells and mouse model of Parkinson's disease (PD), respectively. Gastrodin significantly and dose dependently protected dopaminergic neurons against neurotoxicity through regulating free radicals, Bax/Bcl-2 mRNA, caspase-3, and cleaved poly(ADP-ribose) polymerase (PARP) in SH-SY5Y cells stressed with MPP^+^. Gastrodin also showed neuroprotective effects in the subchronic MPTP mouse PD model by ameliorating bradykinesia and motor impairment in the pole and rotarod tests, respectively. Consistent with this finding, gastrodin prevented dopamine depletion and reduced reactive astrogliosis caused by MPTP as assessed by immunohistochemistry and immunoblotting in the substantiae nigrae and striatata of mice. Moreover, gastrodin was also effective in preventing neuronal apoptosis by attenuating antioxidant and antiapoptotic activities in these brain areas. These results strongly suggest that gastrodin has protective effects in experimental PD models and that it may be developed as a clinical candidate to ameliorate PD symptoms.

## 1. Introduction

Parkinson's disease (PD) is neurodegenerative disorder characterized by progressive loss of dopaminergic neurons in the pars compacta of the substantia nigra (SNpC), which leads to clinical symptoms of rigidity, resting tremor, and bradykinesia [1, 2]. 1-Methyl-4-phenyl-1,2,3,6-tetrahydropyridine (MPTP) is a known mitochondrial complex I inhibitor that selectively damages dopaminergic neurons in the SNpC and leads to the depletion of dopamine in the striatum resulting in parkinsonian syndrome [1, 3, 4]. Following parenteral administration, MPTP enters the brain and is metabolized to 1-methyl-4-phenylpyridinium (MPP^+^) by monoamine oxidase-B, which induces neuronal cell death [5]. The subchronic MPTP mouse model is quite a popular regimen, as it causes apoptosis and depletes striatal dopamine by 40–50% in young adult C57BL/6 mice, and dopaminergic lesions stabilize within 21 days after MPTP administration [6, 7]. Human dopaminergic SH-SY5Y cells possess many of the qualities of human neurons and, as such, have served as a well-established PD model [8, 9].

Synthetic drugs cause undesirable adverse effects, whereas natural products are considered safe and effective. Herbal medicines are becoming popular for improving quality of life with either limited or no side effects [10]. The influence of natural products is quite marked in drug discovery. Of 1,335 approved drugs from the 1940s to date, 59 (4%) were derived from natural products, and 299 (22%) were derived from a natural product (semisynthetic modification) [11]. The phenolic glucoside gastrodin is an active constituent of *Gastrodia elata* Blume (Orchidaceae) and is one of the most important traditional plants in Oriental countries. Gastrodin has been used to treat various ailments such as headache, dizziness, vertigo, and convulsive illnesses in traditional medicine. Apart from traditional claims, scientific reports support the antioxidative [12, 13], anticonvulsive [14], anti-inflammatory [15–17], antiepileptic [18], antiobesity [19], anxiolytic [20], and learning and memory improvements [21, 22] in activities of gastrodin. Gastrodin significantly attenuates the expression levels of neurotoxic proinflammatory mediators including inducible nitric oxide synthase, cyclooxygenase-2, and the proinflammatory cytokines tumor necrosis factor-*α* and interleukin-1*β* in lipopolysaccharide-stimulated microglial cells [17, 23]. Gastrodin is also a potent antioxidant and free radical scavenger that decreases the levels of lipid peroxidation [24] and increases the expression of genes encoding antioxidant proteins [25]. In addition, gastrodin has been used in clinics as an effective and safe drug for preventing neurocognitive decline following cardiopulmonary bypass [26] and is beneficial to older patients with refractory hypertension by improving the balance of endothelin and nitric oxide in plasma [27], suggesting safe use in humans. 

However, studies evaluating the neuroprotective role of gastrodin in MPP^+^/MPTP PD experimental models have not been conducted. In the present study, we elucidated the probable neuroprotective mechanism of gastrodin using *in vitro* and *in vivo* experimental models of PD. SH-SY5Y cells were treated with 1 mM MPP^+^ and/or gastrodin (1, 5, and 25 *μ*M) and assessed for changes in cell morphology and cell viability. We further characterized antioxidant (reactive oxygen species (ROS) inhibition, superoxide dismutase (SOD) activity) and antiapoptotic activities (Bax/Bcl-2 mRNA, caspase-3, and cleaved PARP) in SH-SY5Y cells stressed with MPP^+^ to elucidate the neuroprotective effects of gastrodin. Furthermore, we used MPTP-intoxicated C57BL/6 mice and then carried out behavioral (pole and rotarod tests), antioxidant (SOD, heme oxygenase-1 (HO-1)), histochemical (tyrosine hydroxylase (TH) and glial fibrillary acidic protein (GFAP)), and histobiological evaluations (Bax, Bcl-2, cleaved poly(ADP-ribose) polymerase (PARP), and caspase-3 activity). The present results reveal gastrodin as a potential candidate with neuroprotective and antiapoptotic activities and strongly suggest gastrodin as a clinical candidate for PD.

## 2. Materials and Methods 

### 2.1. Reagents

Gastrodin was purchased from PhytoLab (Vestenbergsgreuth, Germany). MPP^+^, MPTP, 2,2-azobis (2-amidinopropane) hydrochloride (AAPH), 1,1-diphenyl-2-picrylhydrazyl (DPPH), (4-pyridyl-1-oxide)-N-tert-butylnitrone, 3-(3,4-dimethylthiazole-2-yl)-2,5-diphenyl-tetrazolium bromide (MTT), and a caspase-3 assay kit were purchased from Sigma-Aldrich (St. Louis, MO, USA). The 10x RIPA buffer was obtained from Millipore (Milford, MA, USA). The protease inhibitor and phosphatase inhibitor cocktail tablets were supplied by Roche (Roche, Indianapolis, IN, USA). Tween 80 was purchased from Merck (Calbiochem, Darmstadt, Germany). Six-well and 96-well tissue culture plates and 100 mm culture dishes were purchased from Nunc Inc. (Aurora, IL, USA). Dulbecco's modified Eagle's medium (DMEM) and fetal bovine serum (FBS) were purchased from Gibco-BRL Technologies (Carlsbad, CA, USA). All other chemicals used in this study were of analytical grade and were obtained from Sigma Chemical Co.

### 2.2. Cell Culture and Treatments

Human dopaminergic SH-SY5Y cells were obtained from the American Type Culture Collection (Manassas, VA, USA) and cultured in DMEM supplemented with 10% (v/v) inactivated FBS, and 100 U/mL penicillin/streptomycin. The cells were maintained at 37°C in 5% CO_2_ and a 95% humidified air incubator for the indicated time. All experiments were carried out 24–48 h after the cells were seeded. The cells were pretreated with various concentrations (1, 5, and 25 *μ*M) of gastrodin for 4 h before incubation in medium containing 1 mM MPP^+^. Control cells were treated with the same medium without drugs.

### 2.3. Animals and Treatments

Six-week-old male C57BL/6 mice were obtained from Samtako Bio Korea (Gyeonggi-do, Korea) and acclimatized before use. Mice ~8 weeks of age and 25–28 g in weight were used in the present study. All experiments were performed in accordance with the Principles of Laboratory Animal Care (NIH publication no. 85-23, revised 1985) and Guidelines for Animal Experiments at Konkuk University. The animals were housed in a controlled environment (23 ± 1°C and 50% ± 5% humidity) and allowed food and water *ad libitum*. The room lights were on between 8:00 h and 20:00 h; sixty animals were divided into five groups containing 12 animals each. The groups included vehicle, MPTP, 10 mg/kg gastrodin, 30 mg/kg gastrodin, and 60 mg/kg gastrodin. Gastrodin was administered perorally for 15 days at the respective doses, and MPTP was administered intraperitoneally for the last 5 days of gastrodin treatment. All groups except the vehicle group received an injection of 30 mg/kg/day MPTP for 5 days. MPTP and gastrodin were dissolved in saline and prepared just prior to dosing. 

### 2.4. Assessment of Cell Viability

Cell viability was measured using the quantitative colorimetric MTT assay, which reveals mitochondrial activity of living cells, as described previously [28]. MTT dissolved in phosphate-buffered saline was added at the end of incubation to a final concentration of 0.5 mg/mL. After 4 h incubation at 37°C and 5% CO_2_, the supernatants were removed, and the formed formazan crystals in the viable cells were measured at 550 nm using a microplate reader (Molecular Devices, Sunnyvale, CA, USA). 

### 2.5. Measurement of Free Radical Scavenging Activity, Intracellular Reactive Oxygen Species (ROS), and Superoxide Dismutase (SOD) Activity

Free radical scavenging activity was evaluated using an electron spin resonance (ESR) spectrometer (JEOL, Tokyo, Japan). DPPH radical scavenging activity was measured using a method described previously [29]. A sample solution of gastrodin was added to 60 *μ*M DPPH in methanol and incubated for 2 min. Alkyl radicals were generated by AAPH. The reaction mixture, containing 10 mM AAPH, 10 mM 4-POBN, and gastrodin of various concentrations in PBS (pH 7.4), was incubated at 37°C in a water bath for 30 min. Hydroxyl radicals were generated by the iron-catalyzed Haber-Weiss reaction (Fenton-driven Haber-Weiss reaction), and the generated hydroxyl radicals reacted rapidly with nitrone spin-trap DMPO. The reaction mixture, containing 0.3 M DMPO, 10 mM FeSO_4_, 10 mM H_2_O_2_, and various concentrations of gastrodin in PBS (pH 7.2), was incubated for 2.5 min. Superoxide radicals were generated by an UV-irradiated riboflavin/EDTA system. The reaction mixture, containing 0.8 mM riboflavin, 1.6 mM EDTA, 0.8 M DMPO, and various concentrations of gastrodin, was irradiated for 1 min under a UV lamp at 365 nm. The ESR spectrum was recorded for each radical using an ESR spectrometer. The intracellular ROS production was measured using a nonfluorescent compound 2′,7′-dichlorofluorescein diacetate (DCFH-DA) in SH-SY5Y cells. It measures the formation of hydrogen peroxide generated by an oxidative metabolic burst. Viable cells can deacetylate DCFH-DA to 2′,7′-dichlorofluorescein (DCFH), which is not fluorescent. This compound reacts quantitatively with oxygen species within the cell to produce a fluorescent dye 2′,7′-dichlorofluorescein (DCF), which remains trapped within the cell and can be measured to provide an index of ROS level. After the drug treatment, cultures were washed with PBS, loaded with 20 *μ*M DCF-DA for 30 min at 37°C, and then washed again with PBS. DCF fluorescence was analyzed using a fluorescence plate reader (Spectramax M2e, Molecular Devices) at excitation and emission wavelengths of 490 and 530 nm. SOD was measured as an additional indicator of change in oxidative mechanism following treatment(s) in SH-SY5Y cells and tissue lysate. SOD was determined using Cayman chemical kit (Ann Arbor, MI, USA) as per the manufacturer's instructions. 

### 2.6. Behavioral Testing: Pole and Rotarod Tests

The pole test for bradykinesia was conducted using a modification of a method reported previously [30]. The mice were positioned with the head up at the top of a rough-surfaced pole (8 mm diameter and 55 cm height), and the T-turn and total locomotor activity (TLA) were measured. The T-turn is the time that the animal takes to completely turn his face downside. TLA is the time until the mouse arrives on the floor. The duration of these parameters reflects bradykinesia. This test was performed five times successively for each mouse, and the average was taken for analysis.

The rotarod test was performed as described previously [31] with slight modifications. One week after the last MPTP injection, rotarod performance was evaluated on the suspended rod of an accelerating rotarod apparatus (diameter: 3 cm) that accelerates at a constant rate from 1 to 30 rpm for 300 s. Mice were trained for 3 consecutive days, and they were placed on the rod for five trials. Time was recorded for each trial. A trial ended when the mouse fell off the rotarod or after time had reached 300 s. A resting time of 180 s was allowed between each trial. 

### 2.7. Immunohistochemistry

Mice were anesthetized with sodium pentobarbital (50 mg/kg, i.p.) after performing the behavioral experiments and their brains were perfusion-fixed with 4% paraformaldehyde in 0.1 M phosphate buffer (pH 7.4) following a saline flush as described previously [6]. The brains were removed after perfusion fixation at 4°C and immersed in the same fixative and dehydrated in 30% sucrose solution until they were embedded in tissue freezing medium (Leica, Gmbh Heidelberger, Germany). Frozen sections (30 *μ*M) of the striatum and SNpC were used for immunohistochemistry as described previously [32]. Primary antibodies used for TH and GFAP were anti-TH rabbit antibody (1 : 1000; Calbiochem San Diego, CA, USA) and a rabbit polyclonal antibody to GFAP (1 : 5000; Abcam, Cambridge, UK), respectively. The TH immunohistochemistry sections were incubated with biotinylated anti-rabbit (Vector Laboratories, Burlingame, CA, USA) antibody for 1 h, followed by incubation in the avidin-biotin-peroxidase complex Vectastain Elite ABC Kit (Vector Laboratories) for 60 min at room temperature, according to the supplier's recommendations. Last, the sections have reacted with the Vector DAB substrate kit (Vector Laboratories) for color development. The GFAP sections were incubated with rabbit antigoat GFAP antibody (1 : 200) (Alexa Flour 488, Invitrogen, Carlsbad, CA, USA) for 1 h. Stained cells were viewed under a bright field microscope (Carl Zeiss Inc., Oberkochen, Germany). 

### 2.8. Immunoblot Analysis

A 0.1 mL aliquot of RIPA buffer (1x PBS, 1% NP-40, 0.5% sodium deoxycholate, 0.1% SDS containing freshly added protease inhibitor cocktail (Calbiochem)) was added to cells cultured in 100 mm plates to obtain the total cell lysate. Cells were scraped, incubated for 10 min on ice, and centrifuged at 14,000 ×rpm for 10 min at 4°C. For the animal experiment, tissues were washed two times with PBS, placed at 4°C, and homogenized using a 1 mL syringe in lysis buffer (1x RIPA lysis buffer, protease inhibitor cocktail, and phosphatase inhibitor cocktail) and then finally passed through a 31^1/2^ gauge syringe needle and centrifuged at 14,000 rpm at 4°C for 15 min. The supernatants were collected for further analysis. Protein concentration was determined by the Bio-Rad DC Protein Assay (Hercules, CA, USA). 15 *μ*g of whole cell lysates (cell experiments) or 40 *μ*g of protein (animal experiments) were separated electrophoretically by 10% sodium dodecyl sulfate-polyacrylamide electrophoresis (SDS-PAGE), and the resolved proteins were transferred to polyvinylidene difluoride membranes (Millipore, Bedford, MA, USA). The membranes were incubated for 1 h with 5% skimmed milk in PBS buffer to block nonspecific binding and then incubated with primary antibodies to anti-TH, anti-PARP (1 : 1000; Cell Signaling Technology), anti-GFAP (1 : 30,000; Abcam), anti-HO-1 (1 : 1000; Stressgen biotechnology), and anti-*β*-actin (1 : 2000; Cell Signaling Technology). The blots were visualized using the SuperSignal West Pico Chemiluminescent Substrate Detection System (Thermo Scientific, Rockford, IL, USA), according to the manufacturer's procedure. The optical densities of the antibody-specific bands were analyzed using a luminescent image analyzer, LAS-3000 (Fuji, Tokyo, Japan). 

### 2.9. Total RNA Extraction and Reverse Transcription Polymerase Chain Reaction (RT-PCR)

SH-SY5Y cells (1 × 10^6^ cells/well) were cultured in 6-well plates, and total RNA was isolated by extraction with TRIzol (Invitrogen). Total RNA was also extracted from midbrain and striatal tissues using TRIzol reagent for RT-PCR. A total of 2.5 *μ*g of total RNA was reverse-transcribed using a First Strand cDNA Synthesis Kit (Invitrogen). PCR was performed using the above prepared cDNA as the template. GAPDH was used as the internal control to evaluate relative expression of HO-1, Bcl-2, and Bax. PCR amplification was conducted using specific primers (Bioneer, Daejeon, Korea), as reported previously [32]. The primer sequences are provided in Supplementary Table 1 of the Supplementary Material available online at http://dx.doi.org/10.1155/2013/514095. 

### 2.10. Caspase-3 Activity Assay

Caspase-3 activity was detected using a Colorimetric Caspase-3 Assay kit (Sigma-Aldrich), according to the manufacturer's protocol. Briefly, the reaction mixture (total volume, 200 *μ*L) contained 5 *μ*L of cell lysate/tissue lysate and 5 *μ*L of caspase-3 substrate (Ac-DEVD-pNA; final concentration, 200 *μ*M) in assay buffer, and the assay was carried out in a 96-well plate. A control reaction mixture contained 5 *μ*L of cell lysate and 5 *μ*L of the specific caspase-3 inhibitor (Ac-DEVD-CHO; final concentration, 20 *μ*M) in assay buffer and was used to account for nonspecific hydrolysis of the substrate. Both mixtures were incubated for 90 min at 37°C, and emission and excitation absorbance wavelengths of 360 and 460 nm, respectively.

### 2.11. Statistical Analyses

All data were analyzed using Graph Pad Prism version 5.01 (Graph Pad, Inc., La Jolla, CA, USA). All data are expressed as mean ± standard error of at least three independent experiments performed in triplicate in the case of the *in vitro* or histological and histobiochemical experiments. The comparison of interest in the behavioral experiment was the effect of each treatment compared to control mice and MPTP-intoxicated mice versus gastrodin and MPTP intoxicated mice. The statistical analysis was performed with a one-way analysis of variance followed by Tukey's multiple comparison test. *P* values < 0.05 were considered statistically significant.

## 3. Results

### 3.1. Gastrodin Ameliorates MPP^+^-Induced Loss of Neuronal Cell Viability

**Figure 1:**
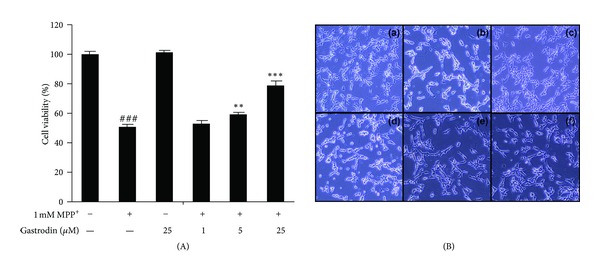
Effect of gastrodin on MPP^+^-induced neurotoxicity in the dopaminergic neuroblastoma SH-SY5Y cell line. Cells were exposed to gastrodin and 1 mM MPP^+^ for 48 h. Cell viability (A) and morphology of SH-SY5Y cells (B) after treatment with control (a), 1 mM MPP^+^ (b), 25 *μ*M gastrodin (c), 25 *μ*M gastrodin + 1 mM MPP^+^ (d), 5 *μ*M gastrodin + 1 mM MPP^+^ (e), and 1 *μ*M gastrodin + 1 mM MPP^+^ (f). Data are percentages of values in the untreated control cultures and are means ± standard errors of three independent experiments in triplicate. ^###^ *P* < 0.001 compared with the control group, **P < 0.01, and ***P < 0.001 compared with the MPP^+^-treated group (one-way analysis of variance followed by Tukey's post hoc test).

Treatment of SH-SY5Y cells with 1 mM MPP^+^ alone for up to 48 h resulted in marked cell death as evaluated by the MTT assay. Cell viability improved significantly in a dose-dependent manner when the cells were pretreated with various concentrations of gastrodin (1, 5, and 25 *μ*M) for 4 h prior to adding 1 mM MPP^+^. Gastrodin alone did not cause any significant cytotoxicity. When cell viability in serum-free conditions was defined as 100% survival, viability of cells treated with 1 mM MPP^+^ decreased to 50.5 ± 1.4%. The viability of cells incubated with 1, 5, and 25 *μ*M gastrodin was 51.7 ± 3.1%, 59.4 ± 1.0%, and 78.2 ± 3.2% of the control, respectively (Figure 1(A)). SH-SY5Y cells treated for 48 h with 1 mM MPP^+^ showed morphological changes normally associated with cell death such as cell shrinkage and rounding up of cell bodies (Figure 1(B)). The MPP^+^-induced changes in cell morphology were attenuated by gastrodin. The cells showed no morphological changes when treated with gastrodin alone.

### 3.2. Antioxidant Activity of Gastrodin

**Figure 2:**
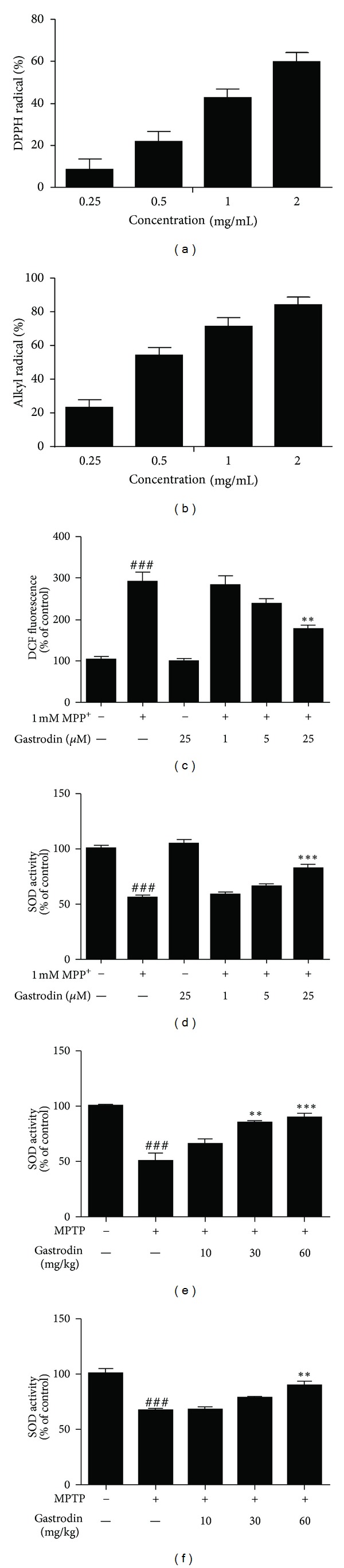
Effect of gastrodin on DPPH (a) and alkyl (b) free radical scavenging activities. Gastrodin prevented the ROS generation in 1 mM MPP^+^-treated SH-SY5Y cells as measured by fluorometric analysis using DCFH-DA (c). Gastrodin augmented the MPP^+^- and MPTP-induced perturbation in superoxide dismutase (SOD) activity in SH-SY5Y cells (d), striatum (e), and SNpC (f), respectively. Data are percentages of values in the untreated control cultures and are means ± standard errors of three independent experiments in triplicate. ^###^ *P* < 0.001 compared with the control group, **P < 0.01, and ***P < 0.001 compared with the MPP^+^-treated group (one-way analysis of variance followed by Tukey's post hoc test).

The potential for gastrodin to quench free radicals such as DPPH, alkyl, hydroxyl, and superoxide radicals was investigated using ESR spectroscopy. DPPH is a stable free radical, accepts an electron or hydrogen radical to become a stable diamagnetic molecule, and has been used to evaluate free radical scavenging activity of natural antioxidants. The capacity of gastrodin to scavenge DPPH was measured by ESR spectrometry. DPPH radical scavenging activity of gastrodin increased in a dose-dependent manner with an IC_50_ value of 1.32 ± 0.04 mg/mL (Figure 2(a)). The alkyl radical spin adduct of the 4-POBN/free radical was generated from AAPH at 37°C for 30 min, and a decrease in the ESR signals was observed with increasing gastrodin doses. Alkyl radical scavenging activity of gastrodin (0.25, 0.5, 1, and 2 mg/mL) was 22.96%, 53.81%, 71.36%, and 83.51%, respectively, with an IC_50_ value of 0.49 ± 0.01 mg/mL (Figure 2(b)). Gastrodin produced a weak nonsignificant scavenging effect on hydroxyl and superoxide radicals (data not shown). These results provide firm evidence of the significant antioxidant effects of gastrodin, particularly on DPPH and alkyl radical scavenging activities. We measured ROS generation in 1 mM MPP^+^-treated cells by fluorometric analysis using DCFH-DA. Cells exposed to MPP^+^ displayed an obvious increase in DCF fluorescence at time-dependent when compared to the control cultures. The DCF fluorescence in cells exposed to 1 mM MPP^+^ at 24 h was 290.4 ± 40.6% higher than that of the control group. However, treatment with gastrodin effectively reduced the ROS generation and the suppressing effects strengthened with the increase of concentration of gastrodin (Figure 2(c)). Furthermore, we investigated SOD activity in MPP^+^/MPTP model in SH-SY5Y cell and mouse, respectively. MPP^+^/MPTP displayed perturbations in the activities of SOD. Pretreatment with gastrodin augmented the activities of SOD in the SH-SY5Y cells (Figure 2(d)), striatum (Figure 2(e)), and SNpC (Figure 2(f)).

### 3.3. Gastrodin Attenuates MPTP-Induced Neurodegeneration: TH and GFAP Immunoblots and Immunohistochemistry

**Figure 3:**
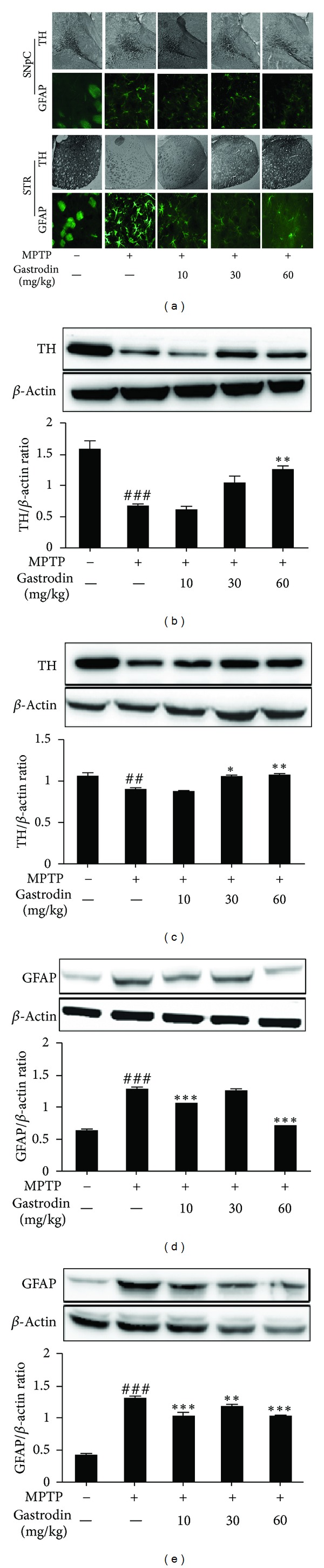
Protective effects of gastrodin against MPTP in the mouse substantia nigra pars compacta (SNpC) and striatum. Gastrodin was administered for 15 days at the respective doses, and MPTP was administered for the last 5 days of gastrodin treatment. All groups except the vehicle group received injections of 30 mg/kg/day MPTP for 5 days. Mice were anesthetized for the immunohistochemical study 7 days after MPTP intoxication and after performing the behavioral experiment. Glial fibrillary acidic protein (GFAP) immunofluorescence and immunohistochemistry for tyrosine hydroxylase (TH) were performed in the SNpC and striatum (a). Gastrodin protected against changes in TH and GFAP expression in the SNpC ((b) and (d)) and striatum ((c) and (e)) after MPTP intoxication. TH and GFAP protein levels in the SNpC and striatum were assessed by Western blot analysis. Bar graphs show quantitative data for TH and GFAP signals that are normalized to the *β*-actin signal (*n* = 4-5 per group). Values are mean ± standard error (^##^ *P* < 0.01, ^###^ *P* < 0.001 versus vehicle group) and (*P < 0.05, **P < 0.01, and ***P < 0.001 versus MPTP group).

TH, a marker of dopamine activity and GFAP, an astrocyte protein marker, were evaluated. The GFAP protein plays a significant role in the interactions of astrocytes with other cells that are required for the formation and maintenance of myelin. Additionally, GFAP may help to maintain the protective blood brain barrier that can only be crossed by certain solutes of 50–52 kDa [33]. Due to these factors, GFAP has received much attention as a proposed biomarker in studies of central nervous system diseases [34, 35]. The density of GFAP-positive astrocytes significantly increases and is negatively correlated with the severity of dopamine neuron depletion [36]. Gastrodin restored decreased and increased expression of TH and GFAP, respectively, in MPTP-intoxicated mice (Figure 3(a)). Furthermore, these results were also reflected in immunoblot analysis, and gastrodin was found to be effective in restoring TH levels (Figures 3(b) and 3(c)) and GFAP expression (Figures 3(d) and 3(e)) in SNpC and striatum, respectively. 

### 3.4. Gastrodin Affects Bcl-2 and Bax Expression and Suppresses MPP^+^/MPTP Induced Caspase-3 Activation

**Figure 4:**
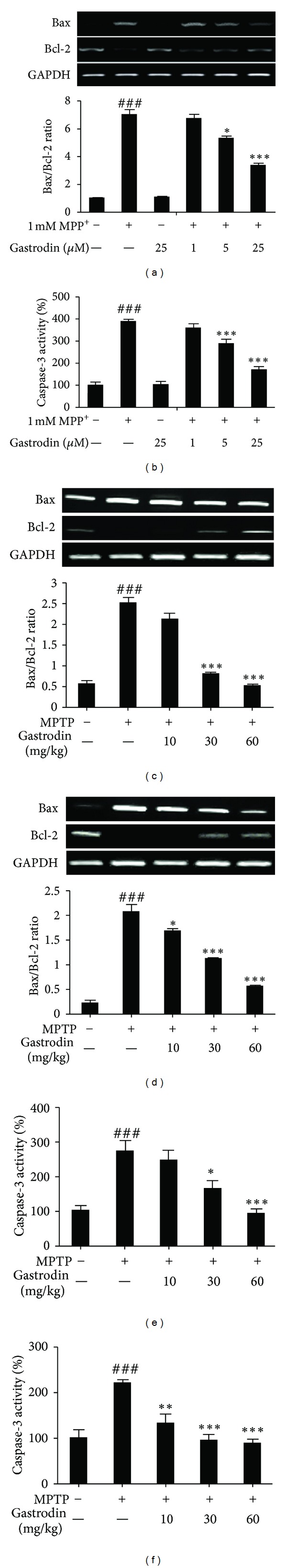
Effects of gastrodin on Bax and Bcl-2 mRNA expression and caspase-3 activity in SH-SY5Y cells and animal tissue treated with MPTP. Bax and Bcl-2 levels were quantified in SH-SY5Y cells by densitometric analysis (a) and in the substantia nigra pars compacta (SNpC) (c) and striatum (d) with their respective Bax/Bcl-2 ratios. Gastrodin inhibited the MPP^+^- and MPTP-induced increase in caspase-3 activity in SH-SY5Y cells (b), SNpC (e), and striatum (f), respectively. Data are from three independent experiments performed in triplicate. Values are mean ± standard error (^###^ *P* < 0.001 versus vehicle group) and (*P < 0.05, **P < 0.01, and ***P < 0.001 versus MPP^+^/MPTP group).

One of the main mechanisms involved in the induction of the mitochondrial apoptotic pathway is a decrease in Bcl-2 levels or, alternatively, an increase in Bax levels. The Bcl-2 family plays a pivotal role in cellular apoptotic machinery [37]. Bcl-2 family members are involved in cell death processes; Bcl-2 is an antiapoptotic protein and Bax exhibits proapoptotic activity [37, 38]. We investigated whether gastrodin had an effect on Bcl-2 and Bax expression in MPP^+^-treated cells using expression analysis. As shown in Figure 4(a), Bax expression increased significantly in the MPP^+^-treated group compared with that in control cells, which was consistent with previous studies [39, 40]. However, gastrodin treatment suppressed Bax mRNA expression in a dose-dependent manner. In contrast, the level of Bcl-2 in the MPP^+^-treated group decreased significantly compared with that in control cells, whereas Bcl-2 expression recovered following gastrodin treatment. The Bax/Bcl-2 ratio in cells exposed to 1 mM MPP^+^ increased 5.6 ± 0.1 fold compared to that in the control group, whereas the ratio decreased in a dose-dependent manner in cells pretreated with 1, 5, and 25 *μ*M gastrodin, suggesting that gastrodin shifted the balance from pro- and antiapoptotic members towards cell survival. Gastrodin treatment alone did not significantly alter the Bax/Bcl-2 ratio (Figure 4(a)). Furthermore, we investigated whether gastrodin had an effect on Bax and Bcl-2 expression in MPTP-intoxicated mice using expression analysis. MPTP-intoxicated mice showed increased Bax expression in both the striatum and SNpC, in agreement with a report published previously [41]. However, prophylactic treatment with gastrodin suppressed Bax mRNA expression in the SNpC and striatum in a dose-dependent manner. In contrast, Bcl-2 level in MPTP-intoxicated mice decreased significantly compared with that in the vehicle group, but prophylactic treatment with gastrodin recovered Bcl-2 expression dose dependently. The Bax/Bcl-2 ratio in MPTP-intoxicated mice increased significantly (^###^ *P* < 0.001 versus vehicle group) compared to that in the vehicle group, whereas prophylactic treatment with gastrodin attenuated the Bax/Bcl-2 ratio in a dose-dependent manner (***P < 0.001 at 30 and 60 mg/kg) in the SNpC (Figure 4(c)) and (*P < 0.05 at 10 mg/kg, ***P < 0.001 at 30 and 60 mg/kg) in the striatum (Figure 4(d)), suggesting that the gastrodin pretreatment shifted the balance from pro- and antiapoptotic members towards cell survival. Caspase-3 is a crucial biomarker of neuronal apoptosis that also acts as an apoptotic executor [42]. Treatment with 1 mM MPP^+^ markedly increased caspase-3 activity, but adding gastrodin attenuated MPP^+^-induced caspase-3 expression to 356.53 ± 22.27%, 285.66 ± 24.71%, and 171.12 ± 16.37%, respectively, in SH-SY5Y cells in a dose-dependent manner (Figure 4(b)). Furthermore, caspase-3 activity showed marked increases in the SNpC and striatum of MPTP intoxicated mice (274.82 ± 28.54% and 219.63 ± 10.37%, resp.). Gastrodin pretreatment dose-dependently attenuated MPTP induced caspase-3 activity to 247.40 ± 28.82%, 167.04 ± 21.43%, and 92.23 ± 12.75%, at 10, 30, and 60 mg/kg, respectively, in the SNpC (Figure 4(e)) and to 130.65 ± 21.11%, 96.71 ± 11.03%, and 88.57 ± 7.53%, at 10, 30, and 60 mg/kg, respectively, in the striatum (Figure 4(f)). 

### 3.5. Gastrodin Suppresses MPP^+^/MPTP-Induced PARP Proteolysis

**Figure 5:**
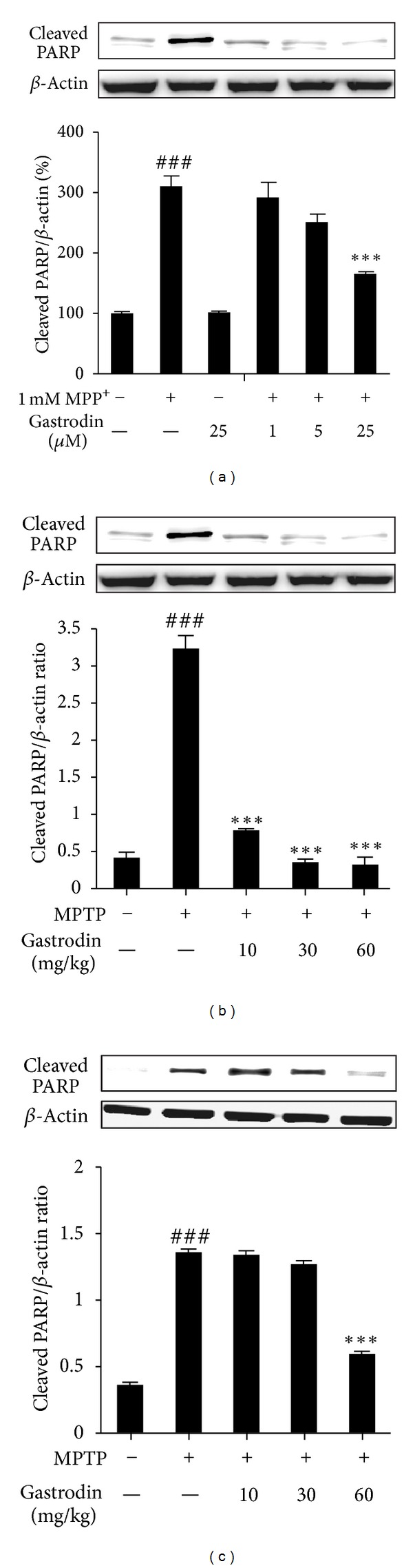
Gastrodin prevents poly(ADP-ribose) polymerase (PARP) cleavage against MPP^+^ in SH-SY5Y cells (a), the substantia nigra pars compacta (SNpC) (b), and striatum (c) after MPTP treatment. PARP protein levels were assessed by Western blot analysis in cells/animal tissues. Bar graphs show quantitative data for PARP signals that are normalized to the *β*-actin signal (*n* = 3-4 per group). Values are mean ± standard error (^###^ *P* < 0.01 versus vehicle group) and (***P < 0.001 and versus MPP^+^/MPTP group).

Caspase-3 also plays a major role in PARP cleavage during early apoptosis in many different cell lines [43, 44]. A previous study reported that MPP^+^ induces increases in PARP proteolysis at 48 h when compared to control cultures [45]. PARP cleavage to an 85 kDa fragment was detected using a polyclonal antibody against full-length PARP (116 kDa), as well as cleaved PARP fragments (85 kDa). PARP proteolysis was enhanced significantly following treatment with 1 mM MPP^+^, but gastrodin concentrations of 1, 5, and 25 *μ*M attenuated MPP^+^-induced PARP proteolysis in a dose-dependent manner (Figure 5(a)). PARP cleavage is another hallmark of apoptosis [44, 46]. Therefore, we further examined PARP cleavage in MPTP-intoxicated mice and the effect of gastrodin on PARP cleavage. MPTP-intoxicated mice showed cleaved PARP (^###^ *P* < 0.001 versus vehicle group), but gastrodin pretreatment attenuated PARP cleavage in the SNpC (Figure 5(b)) (***P < 0.001 versus MPTP group) and striatum (Figure 5(c)) (***P < 0.001 versus MPTP group).

### 3.6. Gastrodin Shows Neuroprotective Effects against MPTP Toxicity in a Mouse PD Model: Pole and Rotarod Tests

**Figure 6:**
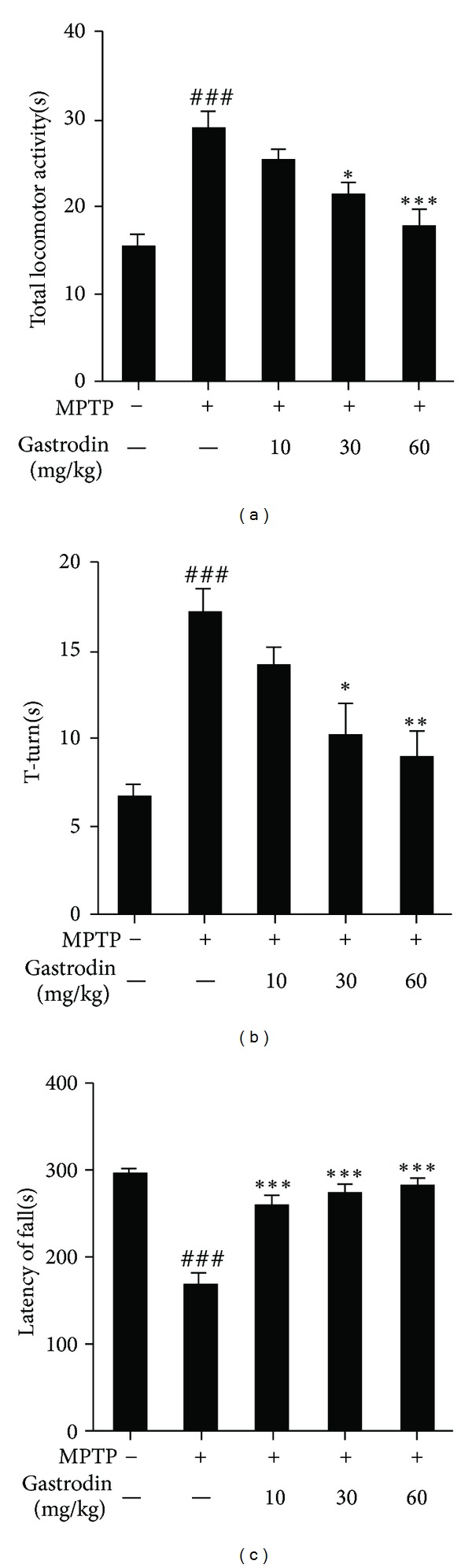
Protective effect of gastrodin against 1-methyl-4-phenyl-1,2,3,6-tetrahydropyridine- (MPTP-) induced behavioral dysfunction in a mouse model of Parkinson's disease. Vehicle or different doses (10, 30, and 60 mg/kg) of gastrodin were administered orally once per day for 15 days, and MPTP (30 mg/kg, i.p.) was injected during the last 5 days. Seven days after the last MPTP injection, the time to arrive at the floor (total locomotor activity) (a) and the time to turn completely downward (T-turn) (b) were recorded with a cutoff limit of 60 s for the pole test. Motor deficit was indicated by the latency of fall (c) during the rotarod test. Values are mean ± standard error (^###^ *P* < 0.001 versus vehicle group) and (*P < 0.05, **P < 0.01, and ***P < 0.001 versus MPTP group).

We performed the pole and rotarod tests to evaluate motor deficits and bradykinesia, respectively, in MPTP-intoxicated mice. The pole test results showed that the T-turn and TLA time were prolonged significantly (^###^ *P* < 0.001 versus vehicle group) after subchronic MPTP treatment. Prophylactic treatment with gastrodin at 30 mg/kg (*P < 0.05 versus MPTP group in the TLA and T-turn) and 60 mg/kg significantly (***P < 0.001, **P < 0.01 versus MPTP group in TLA and T-turn, resp.) shortened the time to reach the platform. The 10 mg/kg gastrodin dose tended to shorten the T-turn and TLA, although the difference was not significant (Figures 6(a) and 6(b)). Thus, gastrodin prevented MPTP-induced bradykinesia. MPTP-intoxicated mice showed decreased performance on the rotarod test (^###^ *P* < 0.001 versus vehicle group), but prophylactic treatment with gastrodin at 10, 30, and 60 mg/kg resulted in improved rotarod test performance (***P < 0.001 versus MPTP group), suggesting that the initial lesions caused by MPTP can be prevented by prophylactic treatment with gastrodin (Figure 6(c)).

## 4. Discussion

In the present study, we provide evidence that gastrodin possessed neuroprotective effects in *in vitro* and *in vivo* PD models possibly by inhibiting oxidative stress and apoptosis-induced neuronal cell death. Oxidative stress is one of the etiologies that could induce neuronal damage in the parkinsonian brain and also modulate intracellular signaling [47]. We used ESR to evaluate free radical scavenging activity of gastrodin. The results showed that gastrodin was a powerful antioxidant with significant radical scavenging activity for DPPH and alkyl radicals. Moreover, the treatment of SH-SY5Y cells with MPP^+^ causes a significant accumulation of intracellular ROS and gastrodin pretreatment ameliorates this effect. In this study, the MPP^+^/MPTP group showed lower SOD activities in SH-SY5Y cells/animals tissue. These results indicate that MPP^+^/MPTP reduce the elimination of hydrogen peroxide and free radicals in the brain. Gastrodin pretreatment in the MPP^+^/MPTP-induced group showed increased SOD activities. These results suggest that the elimination of superoxide anion was enhanced by gastrodin. Furthermore, HO-1, an inducible gene upregulated by oxidative stress, appears to be a component of cellular oxidative stress response [48]. HO-1 is induced by chemicals like MPP^+^/MPTP that produce oxidative stress. HO-1 is a stress responsive enzyme and part of the cell's natural defence mechanisms. In the present study, MPP^+^ caused increased HO-1 expression at protein level in SH-SY5Y cells and gastrodin restored this effect. Furthermore, gastrodin also showed protection against elevated HO-1 level at mRNA and protein levels in SNpC and striatum of MPTP-treated mice (Supplementary Figure 1). 

Generation of ROS causes severe impairment at the cellular level, ultimately leading to neuronal death by apoptosis, which is linked with neurodegenerative disorders such as PD [49]. The Bcl-2 family of intracellular proteins are central regulators of caspase activation and oppose the anti- and proapoptotic members. Commitment to apoptosis in response to diverse physiological cues and cytotoxic agents is governed by Bcl-2 family proteins [37]. Members of the Bcl-2 family, such as Bcl-2, bind to Bax to form Bax : Bcl-2 heterodimers; hence, antagonizing Bax procell death properties [50]. In the present study, MPP^+^ showed a profound effect on the expression of Bcl-2 family members in SH-SY5Y cells. Treatment with gastrodin significantly reduced expression of proapoptotic Bax and significantly increased expression of antiapoptotic Bcl-2 in a dose-dependent manner, thereby ameliorating the increased MPP^+^-induced Bax/Bcl-2 ratio in SH-SY5Y cells. Furthermore, the MPTP mice model provided the opportunity to explore the etiology of PD. Various MPTP regimens can be utilized to screen for possible neuroprotective effects. The subchronic MPTP mouse model is quite a popular regimen that results in apoptosis and depletes striatal dopamine by 40–50% in young adult C57BL/6 mice [6, 7, 51]. Apoptosis-induced neuronal death has been associated with the MPTP subchronic model [7]; accordingly, we determined Bax and Bcl-2 mRNA levels in the SNpC and striatum of MPTP-intoxicated mice. Bax mRNA was upregulated and Bcl-2 mRNA levels decreased in the SNpC and striatum of MPTP-intoxicated mice compared with those in the vehicle group. Prophylactic treatment with gastrodin reduced proapoptotic Bax expression and increased antiapoptotic Bcl-2 expression significantly and dose dependently. 

Of the 12 caspases known in mammals, caspase-3 has been associated with PD neuronal cell death. Caspase-3 is a crucial biomarker of neuronal apoptosis that also acts as an apoptotic executor [42]. PARP, a downstream target of caspase-3, is an abundant nuclear enzyme and is normally involved in DNA repair, but extensive activation of PARP promotes cell death [44, 46]. It is also known that 32-kDa caspase-3 is activated through cleavage into 12-kDa and active 20/17-kDa fragments by apoptotic signals, and that PARP is subsequently cleaved to an 85-kDa fragment [52]. In our study, we found that gastrodin attenuated MPP^+^-induced caspase-3 activation and PARP cleavage in a dose-dependent manner. Furthermore, MPTP-intoxicated mice also showed significant increases in caspase-3 activity in the SNpC and striatum, but various doses of gastrodin prevented caspase-3 activation and thereby might prevent neuronal damage. Gastrodin effectively attenuated MPTP-induced PARP cleavage in a dose-dependent manner, indicating that the protective effect of gastrodin is associated with inhibiting downstream apoptotic signaling pathways, which prevented PARP proteolysis.

Behavioral tests including the pole and rotarod tests were performed after 7 days of MPTP treatment, and mice were sacrificed for brain tissue and expression analyses after MPTP and/or gastrodin treatments. The pole test is a commonly used behavioral test in PD mouse models [53]. Gastrodin at doses of 10, 30, and 60 mg/kg for 15 days attenuated MPTP-induced bradykinesia on the pole test dose dependently. The rotarod test is sensitive to evaluate motor deficits caused by MPTP [31]. In our results, gastrodin protected against motor deficit caused by MPTP. In the histological analysis, TH immunoreactivity decreased in the SNpC and striatum following MPTP treatment, but prophylactic gastrodin treatment significantly reduced MPTP-induced dopaminergic neuronal damage in the SNpC and striatum dose dependently. GFAP is an intermediate astrocyte filament protein. GFAP-positive astrocyte density is negatively correlated with the severity of dopamine neuron depletion [36]. A recent study showed that astrocytes rapidly express GFAP following a subchronic MPTP dose [54]. Astrocytes respond to brain injury through a process termed “reactive gliosis,” in which GFAP regulates the microenvironment, increases neuron survival, and promotes regeneration of neurites and functional recovery of the nervous system [55, 56]. Several studies have shown that astrocytes protect neurons by synthesizing and releasing the free radical scavenger glutathione [57, 58]. In addition, activated astrocytes may stimulate microglial cells, which induce dopaminergic sprouting via the synthesis of neurotrophic factors [59]. MPTP treatment resulted in increased GFAP expression, but gastrodin at doses of 10, 30, and 60 mg/kg prevented gliosis dose dependently as shown in the immunoblot and immunofluorescence analyses. 

Moreover, gastrodin can cross the blood-brain barrier, enter the central nervous system, and protect against nerve lesions [60]. Gastrodin has been used as a safe and effective drug in the clinic for neurocognitive decline and refractory hypertension [26, 27]. Taken together, the similarities between the MPTP model and PD suggest that the neuroprotective effects of gastrodin could be useful against PD and it might be developed as a future clinical candidate for PD.

## Supplementary Material

The respective mouse and human primer sequences of Bax, Bcl-2, HO-1 and GAPDH are provided in Supplementary Table 1
