# Supplementary material for: Gastrodin Protects Apoptotic Dopaminergic Neurons in a Toxin-Induced Parkinson's Disease Model
Source: Evid Based Complement Alternat Med. 2013 Mar 3;2013:514095. doi: 10.1155/2013/514095 (PMC3603713; doi:10.1155/2013/514095)
Supplement: Supplementary file 1 — The respective mouse and human primer sequences of Bax, Bcl-2, HO-1 and GAPDH are provided in Supplementary Table 1 [file 514095.f1.pdf]

Supplementary Table 1. Primers used in this study

Gene Symbol	Primer	
	Forward	Reverse
Bax (m)	CACCAAGGTGCCGGAACTGA	AATGCCCATGTCCCCCAATC
Bcl-2 (m)	ACGACTTCTCCCGCCGCTAC	CCCAGCCTCCGTTATCCTGG
GAPDH (m)	GCAGTGGCAAAGTGGAGATTG	TGCAGGATGCATTGCTGACA
Bax (h)	CTGGACAGTAACATGGAGC	TCTTCTTCCAGATGGTGAGT
Bcl-2 (h)	ACTTTGCAGAGATGTCCAGT	CGGTTCAGGTACTCAGTCAT
GAPDH (h)	GCAGTGGCAAAGTGGAGATTG	TGCAGGATGCATTGCTGACA
HO-1 (m)	TCCAGACACCGCTCCTCCAG	GGATTTGGGGCTGCTGCTTTC

Supplementary Figure 1

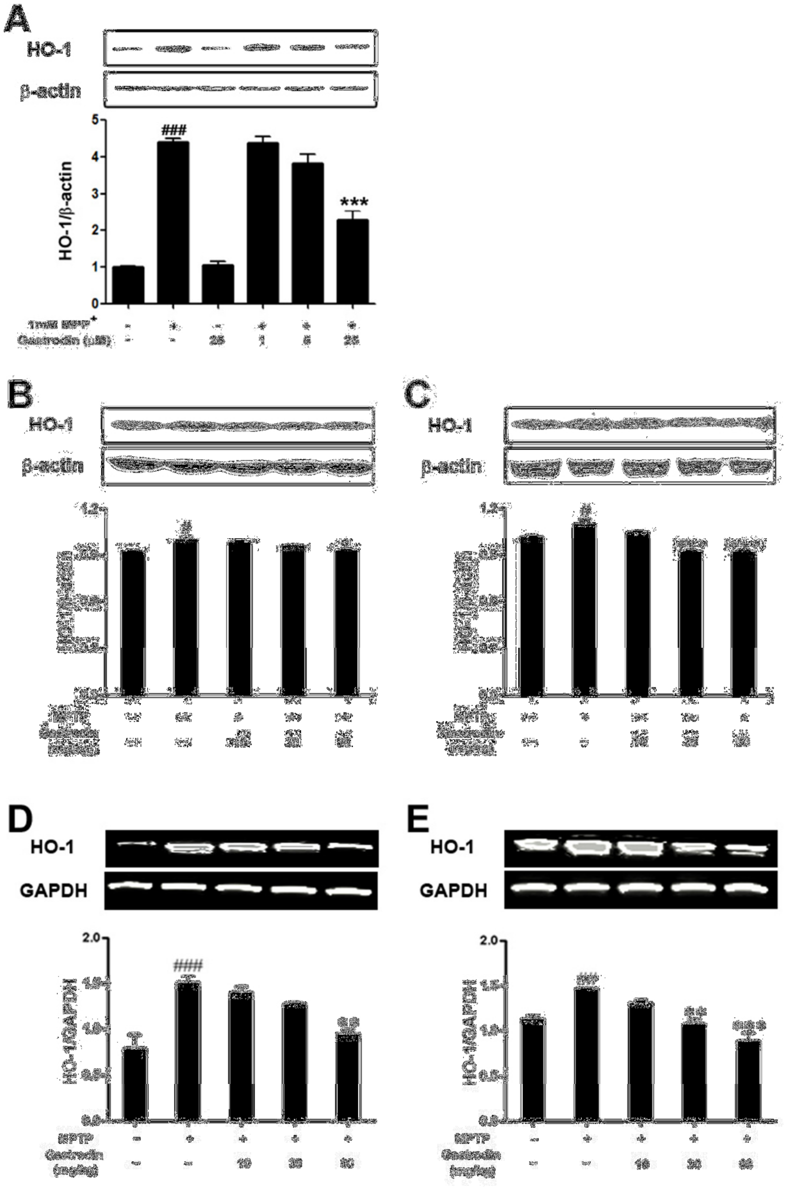

Supplementary Figure 1: Gastrodin prevents MPP^+ and MPTP induced HO-1 expression in SH-SY5Y cells (A), the substantia nigra pars compacta (SNpC) (B), and striatum (C) respectively. HO-1 protein levels were assessed by Western blot analysis in cells/animal tissues. Bar graphs show quantitative data for HO-1 signals that are normalized to the β-actin signal (n = 3–4 per group). Semiquantitative PCR was done in SNpC and striatum area of mice after MPTP treatment. Expression of HO-1 mRNA in

SNpC (D) and striatum (E) Values are mean ± standard error (###$p < 0.01$ vs. vehicle group) and (***$p < 0.001$ and vs. MPP^+/MPTP group)
